# *PDGFRα* Regulated by miR-34a and *FoxO1* Promotes Adipogenesis in Porcine Intramuscular Preadipocytes through Erk Signaling Pathway

**DOI:** 10.3390/ijms18112424

**Published:** 2017-11-15

**Authors:** Yun-Mei Sun, Jin Qin, Shu-Ge Liu, Rui Cai, Xiao-Chang Chen, Xiang-Ming Wang, Wei-Jun Pang

**Affiliations:** Laboratory of Animal Fat Deposition & Muscle Development, College of Animal Science and Technology, Northwest A&F University, Yangling 712100, China; sunyunmei@nwsuaf.edu.cn (Y.-M.S.); qinjin@nwafu.edu.cn (J.Q.); Shugeliu2017@163.com (S.-G.L.); cairui1663@nwsuaf.edu.cn (R.C.); cxca_01@nwsuaf.edu.cn (X.-C.C.); wxmxiaohebei@nwsuaf.edu.cn (X.-M.W.)

**Keywords:** pig, *PDGFRα*, intramuscular fat, adipogenesis, miR-34a, *FoxO1*

## Abstract

Suitable intramuscular fat (IMF) content improves porcine meat quality. The vital genes regulating IMF deposition are necessary for the selection and breeding of an IMF trait. However, the effect and mechanism of *PDGFRα* on IMF deposition are still unclear. Here, *PDGFRα* is moderately expressed in porcine longissimus dorsi muscle (LD), whereas it highly expressed in white adipose tissue (WAT). Moreover, *PDGFRα*-positive cells were located in the gaps of LD fibers which there were IMF adipocytes. Compared with 180-day-old and lean-type pigs, the levels of *PDGFRα* were much higher in one-day-old and fat-type pigs. Meanwhile the levels of *PDGFRα* gradually decreased during IMF preadipocyte differentiation. Furthermore, *PDGFRα* promoted adipogenic differentiation through activating Erk signaling pathway. Based on *PDGFRα* upstream regulation analysis, we found that the knockdown of *FoxO1* repressed lipogenesis by downregulating *PDGFRα*, and miR-34a inhibited adipogenesis through targeting *PDGFRα*. Collectively, *PDGFRα* is a positive regulator of IMF deposition. Therefore, we suggest that *PDGFRα* is a possible target to improve meat quality.

## 1. Introduction

The content of intramuscular fat (IMF) is considered as a crucial indicator of porcine meat quality [1]. Increasing the IMF content can improve the flavor and eating quality of meat [2]. The number of IMF progenitors is associated with the IMF deposition [3]. It is well-known that adipocytes are derived from mesenchymal stem cells (MSCs), which commit into adipocyte lineage and finally give rise to preadipocytes [4,5,6]. The preadipocytes, which have lost the ability to differentiate to other cell lineages, are positive for mesenchymal stem cell markers and negative for blood and endothelial cell markers [7]. Then, preadipocytes go through terminal differentiation to mature adipocytes. The cell surface factors CD24, CD34 and Sca1 are used to isolate and analyze adipocyte precursors from white adipose tissue (WAT) through fluorescence-activated cell sorting (FACS) in mice or humans [5,8]. Otherwise, the biomarker of IMF adipocytes is still unclear.

Platelet-derived growth factor receptor α (*PDGFRα*) is important for embryonic organogenesis and development by effecting the differentiation and function of specialized mesenchymal cells [9]. In the human heart, *PDGFRα* positive cells are cardiac progenitors, which contribute predominantly to the mesenchymal compartments and vascular [10]. Because *PDGFRα* expressed in adipogenic stromal cells of adipose tissues, it has been implicated in the adipocyte lineage [11]. In skeletal muscle, the fat and muscle do not originate from the same progenitor cell [12]. Pericytes are thought to act like MSCs [13,14], which express *PDGFRα* and may contribute to fat deposition in skeletal muscle [15]. In contrast, skeletal muscle satellite cells are committed solely to myogenesis [16]. Another group reported that the PDGFRα-positive mesenchymal progenitors were located in the muscle interstitium, which are the major contributors to IMF deposition in skeletal muscle of mice [17]. Therefore, *PDGFRα* may be a potential cell surface biomarker of IMF preadipocyte progenitors. However, the effect and regulatory mechanism of *PDGFRα* on IMF deposition is still unclear.

MiRNAs (MicroRNAs) are non-coding RNAs, approximately 18–22 nucleotides in length, which regulate gene expression at the post-transcriptional level [18]. *PDGFRα* expression can be inhibited by some miRNAs in tumors. For example, miR-34c inhibits lung cancer proliferation, migration and invasion by targeting *PDGFRα/β* [19]; miR-34a affects the growth of pulmonary artery smooth muscle cells in human by targeting *PDGFRα* [20]. *PDGFRα* can also be regulated by miRNAs in nontumor cells [21]. Therefore, we presume that miR-34a may affect *PDGFRα* expression by targeting it during porcine IMF preadipocyte differentiation.

The *FoxO* transcription factors have a wide range of biological functions, including the regulation of cell proliferation, apoptosis, and differentiation [22]. *FoxO* family members are widely expressed in the mammalian organizations. FoxO proteins are responsible for maintaining the basal expression levels of *PDGFRα*, as well as the accumulation of *PDGFRα* under serum starvation condition [23]. Depletion of endogenous *FoxO* proteins in neuroblastoma cells results in a disregulated differentiation phenotype, which can be rescued by *PDGFRα* expression. *PDGFRα* is a critical downstream target gene of the FoxO1 proteins [24]. However, the relationship between *PDGFRα* and *FoxO1* needs further clarification in porcine IMF deposition.

In this study, we elucidated the effect and regulatory mechanism of *PDGFRα* on pig IMF deposition. Therefore, we suggested that *PDGFRα* could serve as a positive regulator associated with meat quality for pig selection and breeding of IMF trait.

## 2. Results

### 2.1. Porcine Tissue Expression Profile of PDGFRα

**Figure 1:**
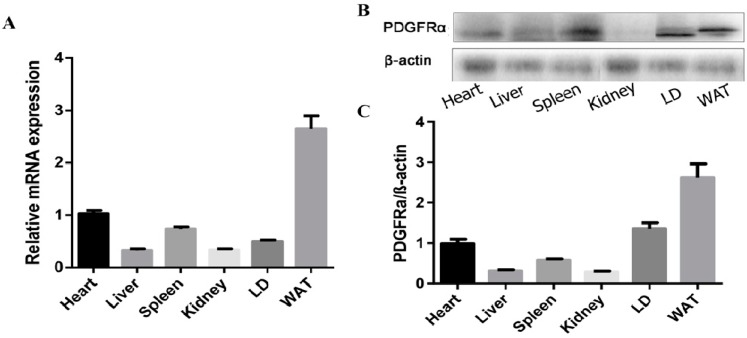
Expression of *PDGFRα* in various tissues of pigs at day 180. (**A**) The levels of *PDGFRα* mRNA by real time qPCR. GAPDH is employed as an internal reference. WAT, white adipose tissue; LD, longissimus dorsi muscle. Each column represents the means of four individual pigs ± SEM; (**B**) detection of *PDGFRα* protein by western blotting. β-actin as loading control; (**C**) the quantify results of protein level. Data represent mean ± SEM, *n* = 4.

To elucidate the tissue expressions of *PDGFRα* in pigs, the levels of its mRNA and protein were detected in various tissues including heart, liver, spleen, kidney, LD and WAT. The results showed that the levels of *PDGFRα*, both mRNA and protein, were abundant in WAT, moderate in LD and the least in the liver (Figure 1A–C).

### 2.2. Identification of PDGFRα-Positive Cells in Porcine LD

**Figure 2:**
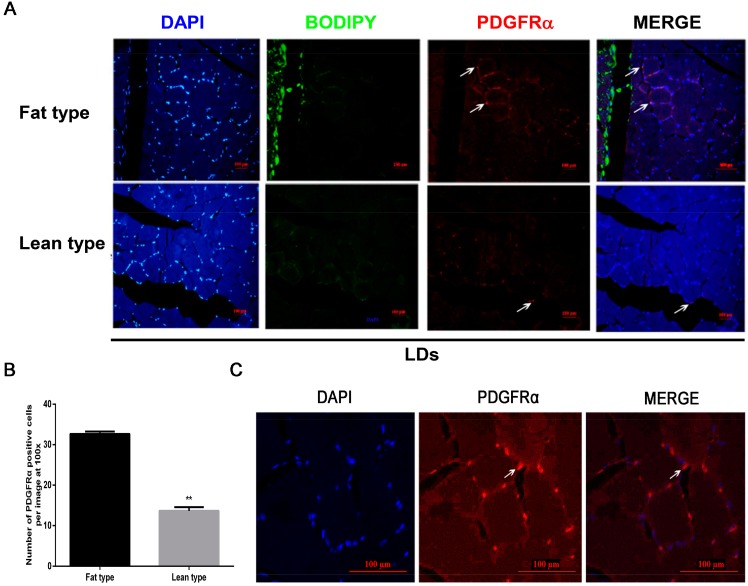

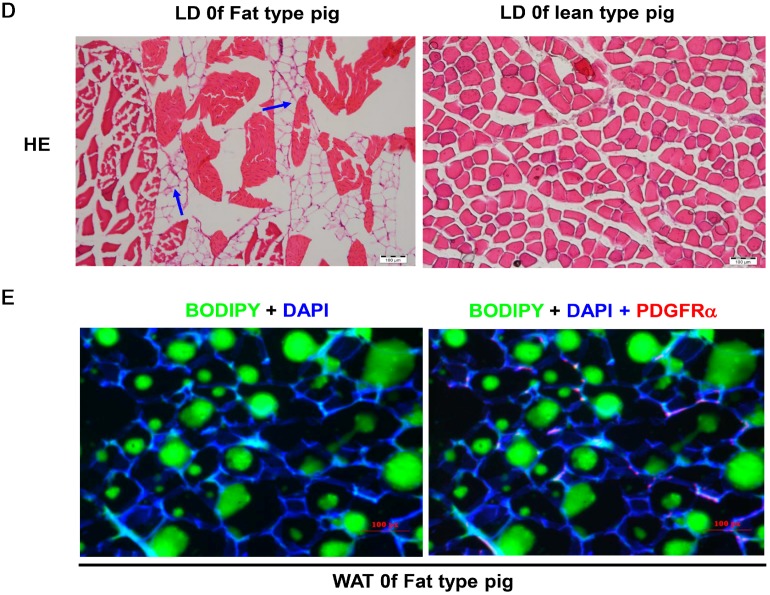
Immunofluorescent staining of fat-type and lean-type pig LD for *PDGFRα*. (**A**) *PDGFRα* indicated red, IMF was green and nuclei stained with DAPI was blue. Scale bar = 100 µm. White arrow indicated *PDGFRα*-positive cells. Three images per section and four sections per pig; (**B**) number of *PDGFRα*-positive cells was counted according to the positive staining for *PDGFRα*; (**C**) localization of *PDGFRα*-positive cell in LD. White arrow indicates a *PDGFRα-*positive cell; (**D**) IMF in LD of fat-type and lean-type pigs. HE, hematoxylin and eosin, blue arrow indicates IMF. (**E**) Subcellular localization of *PDGFRα* in WAT of fat-type pigs at 180-day-old age. WAT, white adipose tissue. Data represent mean ± SEM, *n* = 4. ** *p* < 0.01.

To study a *PDGFRα*-positive cell and its localization in porcine LD, immunofluorescent staining was performed with a *PDGFRα* antibody. The results showed that *PDGFRα*-positive cells were in LD (Figure 2A) and the number of *PDGFRα*-positive cells was significantly more numerous in fat-type pigs than in lean-type pigs (Figure 2B). Interestingly, we further found that *PDGFRα*-positive cells were located in the gaps of LD fibers in which there were IMF adipocytes (Figure 2C). It was clear that the IMF content was greater in LD of fat-type pigs than that of lean-type pigs (Figure 2D). Furthermore, the results indicated that *PDGFRα* was located at mature adipocyte membrane (Figure 2E). Therefore, the above results indicated that *PDGFRα* may be a vital biomarker of pig IMF deposition.

### 2.3. Comparison on the Levels of PDGFRα in Different Ages and Types of Pigs

**Figure 3:**
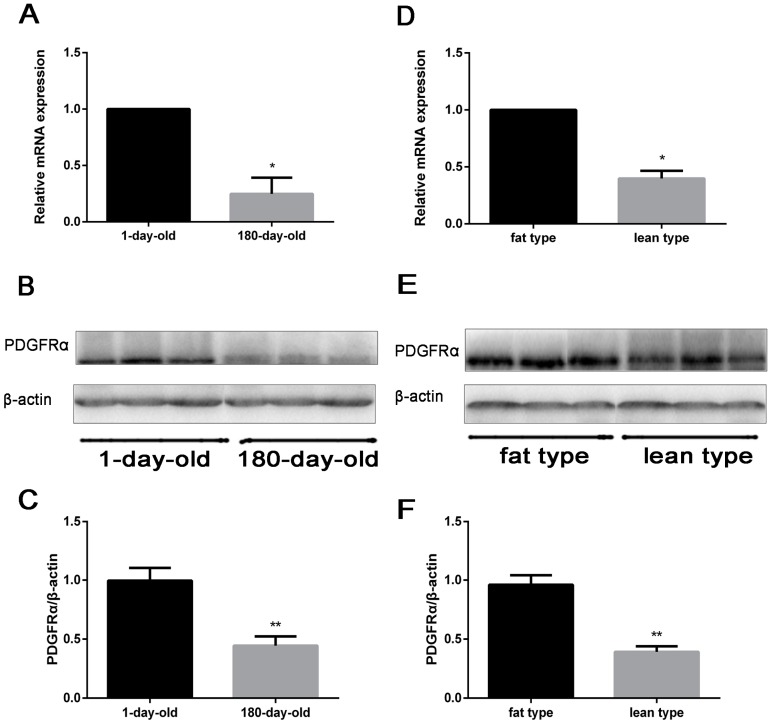
Expression of *PDGFRα* in LD of different age and type pigs. (**A**) The levels of *PDGFRα* mRNA by real-time qPCR. Adult pigs (180 days of age); piglets (1 day of age). *GAPDH* is employed as an internal reference. Each column represents the mean of four individual pigs ± SEM; (**B**) detection of *PDGFRα* protein by Western blotting. β-actin as loading control; (**C**) the quantify protein levels of *PDGFRα* of different age pigs; (**D**) examination of *PDGFRα* mRNA in fat-type and lean-type pigs by real-time qPCR at day 180. Fat-type Bamei pigs; lean-type Yorkshire pigs. β-actin is employed as an internal reference; (**E**) detection of *PDGFRα* by western blotting. β-actin was the loading control. (**F**) The quantify protein levels of *PDGFRα* of different type pigs. Data represent mean ± SEM, *n* = 4. * *p* < 0.05, ** *p* < 0.01.

Because the different day-old and types of pigs is implicated in meat quality, levels of *PDGFRα* mRNA and protein were detected in LD using real time qPCR and western blot. As shown in Figure 3A, the mRNA expression of *PDGFRα* was significantly higher in 1-day-old pigs than that in 180-day-old pigs. Likewise, the protein level was also higher in 1-day-old pigs than that in 180-day-old pigs (Figure 3B,C). Moreover, *PDGFRα* mRNA and protein expressions of fat-type pigs were more abundant than that of lean-type pigs (Figure 3D–F).

### 2.4. Time Course Expression of PDGFRα during Porcine IMF Preadipocyte Differentiation

**Figure 4:**
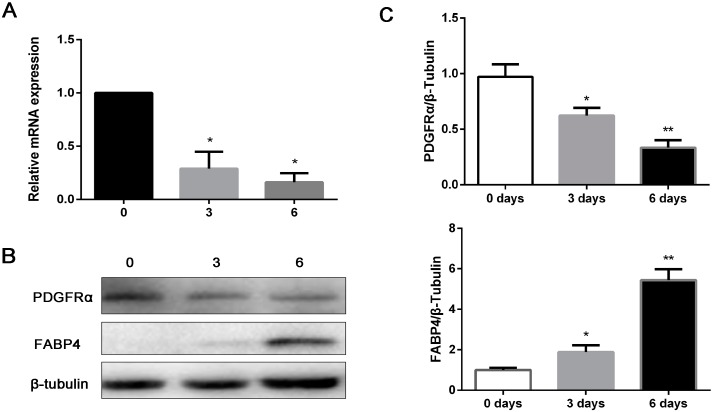
The time-course expression of *PDGFRα* during porcine IMF preadipocyte differentiation. (**A**) The levels of *PDGFRα* mRNA by real time qPCR. *GAPDH* is employed as an internal reference; (**B**) detection of PDGFRα and FABP4 proteins by western blotting. β-tubulin was the loading control; (**C**) the quantify results of protein level. Data represent mean ± SEM, *n* = 4. * *p* < 0.05, ** *p* < 0.01.

To verify whether *PDGFRα* is a potential molecular regulator of IMF deposition, porcine IMF preadipocytes were isolated and cultured using differential adhesion method, and induced to adipogenic differentiation. The expression profile of FABP4 confirmed that IMF preadipocytes differentiated into mature adipocytes (Figure 4B,C). As expected, the levels of *PDGFRα* gradually decreased during IMF preadipocytes differentiation (Figure 4A–C).

### 2.5. Knockdown of Modulated-FoxO1 PDGFRα Inhibits Adipogenesis through Erk Signaling Pathway

**Figure 5:**
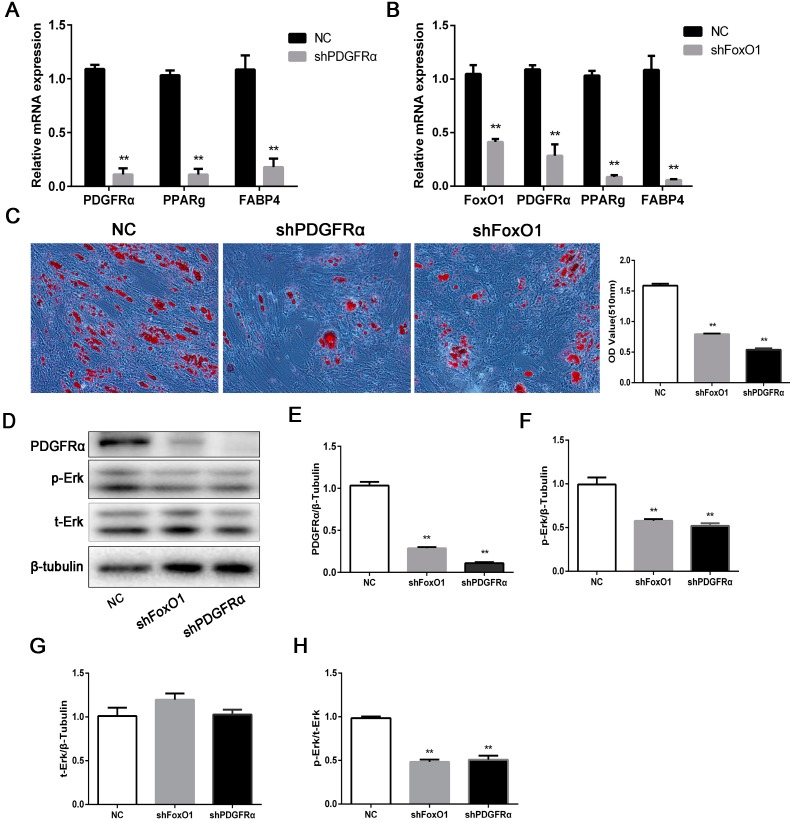
Knockdown of modulated-*FoxO1 PDGFRα* inhibits adipogenesis through Erk signaling pathway. For transfection followed by adipogenic differentiation, cell density must reach 80–85% to ensure that the cells can grow to confluency in two days after transfection. (**A**,**B**) Real-time qPCR was used to detect *PDGFRα*, *FoxO1* and cell differentiation genes, *PPARg* and *FABP4* after 48 h differentation. *GAPDH* is employed as an internal reference; (**C**) oil Red O staining. Scale bar = 100 µm; (**D**) western blot analysis of PDGFRα, Erk and p-Erk. β-tubulin was the loading control. (**E**–**H**) The quantify results of protein level. Data represent mean ± SEM, *n* = 4. ** *p* < 0.01.

To explore the effect and mechanism of *PDGFRα* on IMF deposition, the knockdown experiments of *PDGFRα* and *FoxO1* were performed in porcine IMF preadipocytes. The results showed that knockdown of *PDGFRα* significantly downregulated the mRNA levels of *PDGFRα*, *PPARg* and *FABP4* (Figure 5A). Likewise, knockdown of *FoxO1* markedly reduced the mRNA levels of *PDGFRα*, *PPARg* and *FABP4* (Figure 5B). It implied that *FoxO1* was an upstream regulator of *PDGFRα*. Oil Red O assay revealed that shPDGFRα and shFoxO1 inhibited adipogenesis, respectively (Figure 5C). To determine whether Erk signaling pathway is involved in lipogenesis regulated by *PDGFRα*, the levels of Erk1/2 and p-Erk1/2 were examined. The results showed that *PDGFRα* knockdown repressed Erk1/2 activation by downregulating the levels of p-Erk1/2 (Figure 5D,E–H). Based on above results, we suggested that *PDGFRα*, which was regulated by *FoxO1*, promoted lipogenesis through Erk signaling pathway in porcine IMF preadipocyte differentiation.

### 2.6. miR-34a Represses Lipogenesis through Targeting PDGFRα

**Figure 6:**
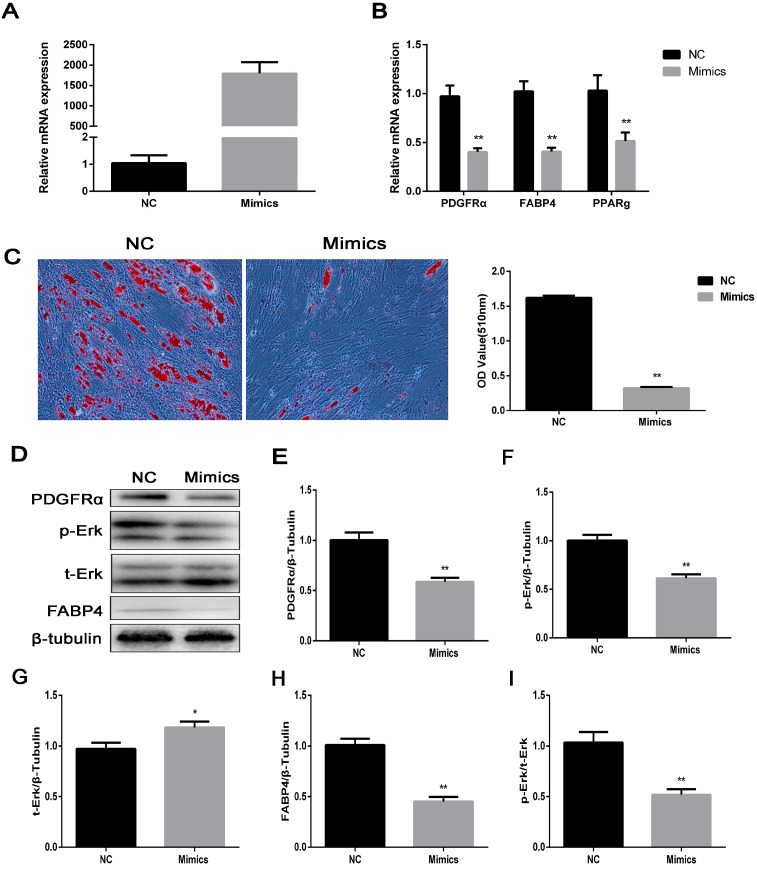
*Mir-34a* targeting *PDGFRα* represses lipogenesis in IMF preadpocytes. (**A**) The overexpression efficiency of *miR-34a* after transfecting *miR-34a* mimics compared with negative control (NC); (**B**) real-time qPCR was used to detect *PDGFRα* and cell differentiation genes, *PPARg* and *FABP4* after 48 h differentiation. *GAPDH* is employed as an internal reference; (**C**) oil Red O staining, scale bar = 100 µm; (**D**) western blot analysis of *PDGFRα* and cell differentiation genes. β-tubulin was the loading control; (**E**–**I**) the quantify results of protein level. Data are presented as mean ± SEM; *n* = 4. * *p* < 0.05, ** *p* < 0.01.

Using mRNA TargetScan analysis, we found that *PDGFRα* may be targeted by *miR-34a*. Therefore, the experiment on *PDGFRα* regulated via *miR-34a* was performed. Overexpression efficiency of miR-34a mimics was high enough to carry out subsequent experiments (Figure 6A). The results showed that overexpression of *miR-34a* dramatically suppressed mRNA levels of *PDGFRα* and lipogenesis genes, including *PPARg* and *FABP4* (Figure 6B), which was consistent with the protein level (Figure 6D,E–I). Furthermore, Oil Red O assay revealed that preadipocyte treated with miR-34a mimics had fewer lipids (Figure 6C). Taken together, *miR-34a* represses lipogenesis in IMF preadipocytes through targeting *PDGFRα*.

## 3. Discussion

Interestingly, *PDGFRα* moderately expressed in porcine LD, but did not express in myotubes [3] implying that *PDGFRα*-positive preadipocytes existed in the LD of pigs. Because *PDGFRα* is an important biomarker of preadipocytes from white adipose tissues [8,25], it is possible that its expression levels are much higher in SAT than in the other tissues. *PDGFRα*-positive cells are the origin of vascular and mesenchymal compartments in the human heart [10], and we also observed that *PDGFRα* was relatively high in the porcine heart. In addition, we found that *PDGFRα* expressed in the LD, WAT, heart, spleen and kidney of the pig, but could hardly be detected in the liver, which may be a reason why obese pigs resist fatty liver disease [26].

Abundant *PDGFRα* expression is a characteristic of undifferentiated MSCs [27]. In addition, it was observed that *PDGFRα* was downregulated after differentiation of mesenchymal progenitors [28]. Consistently, our results indicated that the levels of *PDGFRα* gradually decreased during IMF preadipocyte differentiation in vitro. Many genes are decreased during preadipocyte differentiation, however, they all promote adipogenic differentiation, such as KLF9, KLF4, C/EBPβ, and so on. In our study, the expression of *PDGFRα* was higher at day 0, and it promotes adipogenic differentiation at early phase. It is wondered whether this occurred in vivo as well. Compared with piglets, there was more IMF in the LD of adult pigs [29], but the expression of *PDGFRα* of adult pigs was fewer than that of piglets. We also found that the level of *PDGFRα* is highest in fat-type piglets. Therefore, we thought that *PDGFRα*-positive preadipocytes differentiated into mature adipocytes during IMF deposition with the decrease of *PDGFRα* expression.

Bamei is a local pig breed in China, which is a representative fat-type breed with good IMF content. In contrast, the Yorkshire is an introduced breed, which is regarded as lean-type pig breed [30,31]. Previous studies have shown that fat-type breed pigs are more inclined to IMF deposition than a lean-type pig breed [32] However, whether the number of *PDGFRα*-positive cells is the main cause of the IMF deposition differences between breeds has not been understood. In this study, we found that both *PDGFRα* expression levels and the number of *PDGFRα*-positive cells were significantly greater in fat-type porcine LD than in lean-type porcine LD, suggesting that the *PDGFRα* may be an indicator of IMF deposition in pigs.

Using *PDGFRα* shRNA to knockdown *PDGFRα*, we found that the total Erk1/2 protein level did not change, but the p-Erk1/2 reduced, indicating *PDGFRα*-regulated preadipocyte differentiation through the activation of Erk1/2. The Erk signaling pathway plays a pivotal role in many vital cellular processes, including proliferation and differentiation [33]. Erk1/2 facilitates the early stage of adipogenic differentiation, but needs to be turned off at the adipocyte maturation phase, suggesting a dual role of Erk1/2 in adipogenesis [34]. Our results showed that the knockdown of *PDGFRα* inhibited the activity of p-Erk1/2. The findings suggested that *PDGFRα* mediated the lipogenesis through the Erk signaling pathway in porcine IMF preadipocyte.

To further study the upstream regulation of *PDGFRα* and the knockdown of *FoxO1* using shRNA, the results demonstrated that the knockdown of *FoxO1* markedly reduced the levels of *PDGFRα*. *FoxO1* is always considered as a negative regulator of adipogenesis [35]. However, other research considered that *FoxO1* promoted adipogenesis [36]. In our study, we observed that *FoxO1* promoted lipogenesis during porcine intramuscular preadipocyte differentiation by downregulating *PDGFRα*. The results may be due to other biological processes, of which *FoxO1* contributes. It was reported that silencing *FoxO1* suppressed adipocyte differentiation and LD growth via potently reducing autophagy activity [37]. In addition, using mRNA TargetScan analysis, we found that *PDGFRα* may be targeted by miR-34a. So we transfect miR-34a mimics into porcine IMF preadipocyte. Interestingly, the levels of both *PDGFRα* and p-Erk1/2 were significantly decreased. Therefore, miR-34a may directly target *PDGFRα* and repressed IMF deposition through inhibiting the Erk signaling pathway.

IMF is localized in the interstitial space of muscle tissue [38,39]. In the study, we found that *PDGFRα* may be a membrane protein of porcine IMF adipocytes and a biomarker on IMF content. Based on these results, we suggest that *PDGFRα* may be a candidate gene associated with meat quality for pig selecting and breeding of IMF trait.

## 4. Materials and Methods

### 4.1. Animal and Sample Collection

In this study, all experimental procedures were performed according to the Guide for Northwest A&F University Animal Care Committee. The experimental protocol was approved by the Departmental Animal Ethics Committee of Northwest A&F University (14-233, 10 December 2014).

Fat-type breed Bamei pigs (180 days old) were obtained from Huzhu prefecture of Qinghai province. Lean-type breed Yorkshire adult pigs (180 days old) and piglets (one day old) were provided by the experimental farm of Northwest A&F University. All pigs were killed at a slaughterhouse under the guidelines of Northwest A&F University Animal Care Committee. The heart, liver, spleen, kidney, LD and SAT were dissected, and rinsed with PBS. Samples for western blot and real-time qPCR were frozen in liquid nitrogen until analysis. For the frozen section, the samples were fixed with 4% paraformaldehyde and kept at room temperature until use.

### 4.2. Cell Isolation and Culture

The LD of piglets were separated sterilely, visible connective tissue was removed, then finely minced. Preadipocytes were isolated based on previously studies. In brief, muscle tissues were digested in a digestion buffer consisting of 0.2% collagenase I (270 U/mg; Gibco, Carlsbad, CA, USA) and medium/F12 (DMEM/F12) (Gibco BRL Co., LTD, San Francisco, CA, USA) in a shaking water bath for 1.5 h at 37 °C. The digest sample was filtered aseptically through 70 and 200 µm nylon mesh filters to isolate cells. Then the filtered cells were washed three times with DMEM/F12 by centrifugation at 1500 rpm for 5 min. Cells were seeded at a density of 2.5 × 10^5^ cells per 35-mm culture dish in DMEM/F12 medium with 10% fetal bovine serum (Hyclone, Logan, UT, USA) supplement with penicillin (100 U/mL) and streptomycin (100 U/mL). After 1 h, cells were rinsed with DMEM/F12 medium to wipe off unadhered cells. At day 2, and after cells reach to 100% confluence, cells were cultured with DMEM/F12, supplemented with 5 μg/mL (872 nM) insulin, 1 μM dexamethasone and 0.5 mM isobutyl methylxanthine (IBMX, Sigma-Aldrich, St. Louis, MO, USA), for 2 days to induce differentiate. Then the medium was changed by DMEM/F12 with 10% FBS and 5 μg/mL insulin for another 4 days to maintain differentiation. At day 0, 3 and 6, cells were harvested for further analysis.

### 4.3. Real-Time Quantitative PCR

To detect the expression of miRNA and genes associated with adipogenic differentiation, the total RNA was extracted with Trizol reagent (TakaRa, Otsu, Japan). The concentration of total RNA was measured by the NanoDrop 2000 (Thermo, Waltham, MA, USA). Then we used reverse transcription kits (TakaRa, Otsu, Japan) to synthesize cDNA. For miRNA analysis, specific reverse transcription primers and procedures were used, whereas the normal process was performed for mRNA analysis. In real-time quantitative PCR, every reaction performed in triplicate using SYBR green kits on a Bio-Rad iQTM5 system. The expressions of all genes were normalized to GAPDH, but U6 small RNA was internal reference when examined the level of miR-34a. The primer sequences used for qPCR were shown in Table 1.

### 4.4. Western Blots

The various tissue samples of the pigs and Cells were split by radio immunoprecipitation assay (RIPA) buffer (Beyotime, China) add with protease inhibitor (Pierce, WA, USA). The total protein sample was pointed into and separated in the SDS-polyacrylamide gel. Then transferred it into a PVDF membrane (Millipore, Bedford, MA, USA). Nest, the membrane was blocked in 5% defatted milk for 2 h. After that, the membrane was incubated with primary antibodies at 4 °C overnight followed by a secondary antibody at room temperature for 1.5 h. Protein bands were exposure by chemiluminescence reagents (Millipore, Bedford, MA, USA) and quantified using the Image Lab Image Document. Following primary antibodies were used: *PDGFRα* (1:300; Boster, Wuhan, China), FABP4 (1:500; Santa Cruz Biotechnology, Dallas, TX, USA), β-actin (1:1000; Santa Cruz Biotechnology, Dallas, TX, USA), β-tubulin (1:500; Santa Cruz Biotechnology, Dallas, TX, USA). The secondary antibodies were anti-rabbit, anti-goat and anti-mouse antibodies (Santa Cruz Biotechnology, Dallas, TX, USA). The targeted proteins were detected using the Gel Doc XR System (Bio-Rad, Hercules, CA, USA) as the instructions of the manufacturer.

### 4.5. Frozen Section and HE Staining

Fixed tissues were dehydrated in 30% sucrose and sectioned (10 µm) through sliding microtome (leica, Wetzlar, German). Hematoxylin-eosin staining was realized as previously reported (Godwin, 2011). Then, the sections were observed and took pictures with microscope (Olympus, New York, NY, USA) in 100× magnification.

### 4.6. Immunohistochemistry

Frozen sections were treated with 3% H_2_O_2_ for 10 min to eliminate endogenous oxidase activities and washed three times with PBS. Then, sections were treated with 0.5% TritonX-100/TBS at room temperature for 10 min, and washed three times using distilled water. Antigens were unmasked by high-temperature antigen retrieval (10 min boiling in 0.05 mM Tris/EDTA buffer (pH = 9) and cooling slowly at room temperature. They were then washed three times with TBS. The 5% BSA/TBS as a blocking solution was incubated for 2 h at room temperature. Sections were incubated with primary antibodies of *PDGFRα* (1:100), and diluted in blocking solution at 4 °C overnight. They were then washed three times with TBST and incubated with anti-rabbit red fluorescent secondary antibody (1:1000) (life Technologies, Carlsbad, CA, USA) for 1 h. Then sections were washed with TBST three times for 5 min each. For nuclear visualization, DAPI (4′,6-diamidino-2-phenylindole; Roche, Basel, Switzerland) was incubated 10 min, then the section was rinsed with TBS. After treatment, the sections were observed under a fluorescence microscope (Nikon, Tokyo, Japan).

### 4.7. Oil Red O and BODITY Staining

After being fixed in 4% paraformaldehyde solution, incubated with 0.5% Oil Red O for 30 min, and washed three times with PBS, the myoblast cells were visualized by phase-contrast microscopy (IS-Elements software, Nikon ECLIPSE, Tokyo, Japan). Oil Red O dissolved in lipid droplets was extracted with 100% isopropanol and its relative concentrations were determined by measuring the absorbance at 510 nm.

Frozen sections were treated with 0.5% TritonX-100/TBS at room temperature for 10 min and washed three times with TBS. They were then stained with BODITY (1 µg/mL; Life Technologies, Carlsbad, CA, USA) for 10 min; the sections were washed with TBST three times for 5 min each. For nuclear visualization, DAPI (4′,6-diamidino-2-phenylindole; Roche) was incubated for 10 min, then the section was rinsed with TBS. After treatment, the sections were observed under fluorescence microscope (Nikon, Tokyo, Japan).

### 4.8. Vector Construction Interference

Vectors were constructed with lentiviral plasmid pLentiHI (Invitrogen, Carlsbad, CA, USA) and the inserted shRNAs were designed by online Invitrogen RNAi Designer (Available online: https://rnaidesigner.invitrogen.com/rnaiexpress/). *PDGFRα* and *FoxO1* shRNA vectors were constructed. They were annealed and inserted into pLentiHI plasmids at BamHI and XhoI sites, and confirmed by sequencing.

### 4.9. Lentivirus Package and Infection

The pLentiHI-*PDGFRα* shRNAs, *FoxO1* shRNAs or scrambled shRNA (9 μg), combined with 6 μg Δ8.9 packaging plasmid and 9 μg VSVG envelope protein plasmid, were cotransfected into HEK293T packaging cells (2 × 10^5^ cells per well) with the calcium phosphate method. Forty-eight hours after transfection, the supernatant containing virus particles was collected and passed through a 0.45 μm filter to remove cellular debris. When the cells reached 70–80% confluence, the viral suspension of pLentiHI-*PDGFRα* shRNAs, pLentiHI-*FoxO1* shRNAs or scrambled shRNA was added respectively.

### 4.10. Transfection of miRNA Mimics

Preadipocytes were seeded in 12-well or 6-well plates, and miR-34a mimics or negative control (NC) (GenePharma, Shanghai, China) were transfected into cells at 80% density in 50 nM using X-tremeGENE siRNA Transfection Reagent (Roche) and Opti-MEM (Gibco) culture medium according to the manufacturer’s protocol and the culture medium was changed to fresh medium after 24 h. When the cells grew to confluence after transfection, adipogenic differentiation was initiated by switching to differentiation medium.

### 4.11. Statistical Analysis

Statistical analyses were performed using SPSS18.0 software (SPSS Inc., Chicago, IL, USA). Data represented mean ± SEM. Multiple comparisons were performed by one-way ANOVA followed by Dunnett’s tests. *p* < 0.05 was considered to be significant.

## 5. Conclusions

Taken together, our findings indicated that *PDGFRα*-positive cells were porcine IMF adipocyte and *PDGFRα* contributed to IMF deposition. Mechanically, *PDGFRα* regulated by miR-34a and *FoxO1* promotes adipogenensis in porcine intramuscular preadipocytes through activating Erk signaling pathway.

## Figures and Tables

**Table 1:** Primer sequences used in this study.

Gene	Forward (5′–3′)	Reverse (5′–3′)
*PDGFRα*	ACGACCACCACGGCTCTAAT	TTCTTAGCCAAGCATCGGACT
*FoxO1*	GCAAATCGAGTTACGGAGGC	AATGTCATTATGGGGAGGAGAGT
*PPARg*	AGGACTACCAAAGTGCCATCAAA	GAGGCTTTATCCCCACAGACAC
*aP2*	GAGCACCATAACCTTAGATGGA	AAATTCTGGTAGCCGTGACA
*GAPDH*	AGGTCGGAGTGAACGGATTTG	ACCATGTAGTGGAGGTCAATGAAG
